# Widespread Usutu virus outbreak in birds in the Netherlands, 2016

**DOI:** 10.2807/1560-7917.ES.2016.21.45.30391

**Published:** 2016-11-10

**Authors:** JM Rijks, ML Kik, R Slaterus, RPB Foppen, A Stroo, J IJzer, J Stahl, A Gröne, MGP Koopmans, HP van der Jeugd, CBEM Reusken

**Affiliations:** 1 Dutch Wildlife Health Centre (DWHC), Utrecht University, Utrecht, The Netherlands; 2 These authors contributed equally to the work; 3 Veterinary Pathology Diagnostic Centre (VPDC), Division of Pathology, Department of Pathobiology, Utrecht University, Utrecht, The Netherlands; 4 Sovon, Dutch Centre for Field Ornithology, Nijmegen, The Netherlands; 5 Department of Animal Ecology, Institute for Water and Wetland Research, Radboud University Nijmegen, The Netherlands; 6 Centre for Monitoring of Vectors (CMV), National Reference Centre (NRC), Netherlands Food and Consumer Product Safety Authority (NVWA), Ministry of Economic Affairs, Wageningen, The Netherlands; 7 ErasmusMC, Department of Viroscience, Rotterdam, The Netherlands; 8 Vogeltrekstation – Dutch Centre for Avian Migration and Demography (NIOO-KNAW), Wageningen, The Netherlands

**Keywords:** Flaviviridae, outbreaks, vector-borne infections, Usutu virus, common blackbird (Turdus merula), great grey owl (Strix nebulosa)

## Abstract

We report a widespread Usutu virus outbreak in birds in the Netherlands. Viral presence had been detected through targeted surveillance as early as April 2016 and increased mortality in common blackbirds and captive great grey owls was noticed from August 2016 onwards. Usutu virus infection was confirmed by post-mortem examination and RT-PCR. Extensive Usutu virus activity in the Netherlands in 2016 underlines the need to monitor mosquito activity and mosquito-borne infections in 2017 and beyond.

Here we describe the detection of Usutu virus (USUV; genus *Flavivirus*, family *Flaviridae*), a potentially zoonotic mosquito-borne virus, in live birds captured in the Netherlands in April 2016, and the development of an USUV outbreak with mortality in birds first noticed in August 2016. We provide details on pathological findings in common blackbirds (*Turdus merula*; Tm) and great grey owls (*Strix nebulosa*; Sn*)* and give information on the size of the outbreak, as well as on mosquito abundance in 2016.

## Subclinical bird cases

As part of a targeted study looking at potential routes of incursion of arboviruses, live birds have been captured for sample collection since March 2016. USUV RNA was detected in throat swabs from two healthy blackbirds caught near Wageningen (Gelderland Province) in early April, based on RT-PCR detection of two independent USUV genome targets and sequencing of a 214 bp genome fragment generated in a third, pan-flavi RT-PCR [1,2].

## Outbreak in birds

### Outbreak identification (first set of birds)

The first evidence for an outbreak was obtained in the period from 28 August to 13 September 2016, when an increasing number of case reports of disease-associated mortality in blackbirds were put forward through a citizen science-based alerting system (Table 1). In parallel, the number of blackbirds submitted for post-mortem examination in the context of wildlife disease scanning increased. Eighteen blackbirds were submitted in 2016 until 13 September, and among these one (Tm 1) was obtained on 10 August 2016 and 12 (Tm 2–12, plus one autolytic specimen) were obtained from 28 August onwards (Table 1). Tm 1–12 were from 11 different sites.

**Table 1:** Common blackbirds (*Turdus merula*) observed by citizens to die of disease (n = 136) and those submitted for post-mortem examination (n = 115), the Netherlands, 2005–16

Time period	Proportion of dead blackbirds reported to Sovon^a^ with ‘disease’ as the cause of death		Dead blackbirds investigated at DWHC^b^
	Disease/total deaths	%	Number
2005	0/11	0	NA
2006	0/367	0	NA
2007	0/232	0	NA
2008	1/160	1	0
2009	109/473^c^	23	4
2010	1/161	1	12
2011	0/111	0	3
2012	13/388	3	49^d^
2013	1/103	1	18
2014	2/102	2	5
2015	0/120	0	6
2016 until 13 Sep	9/95^e^	9	18^e^

NA: not available.

^a^ Dutch Centre for Field Ornithology, Nijmegen.

^b^ Dutch Wildlife Health Centre, Utrecht (operational in Utrecht from 2008 onwards).

^c^ All reports indicating blackbirds that died of disease were preceded by the press paying attention to the *Trichomonas gallinae* finch epidemic.

^d^ Fourty-seven of the blackbirds were obtained following the reports in the national press on Usutu virus infection in Germany and a press release on 7 October requesting the public to submit dead blackbirds. There was no evidence for Usutu virus infection at the time [1].

^e^ Among these, eight of nine diseased birds reported to Sovon and 12 of 18 submissions to DWHC were obtained during the 16-day window from 28 August to 13 September. These increasing numbers were not triggered by media attention.

During the same period, the deaths of four captive great grey owls (Sn 1–4) were investigated. The deaths occurred between 13 August and 12 September 2016, in three facilities. The post-mortem findings in birds Tm 1–12 and Sn 1–4 are summarised in Table 2.

**Table 2:** Pathological findings in the common blackbirds (Tm 1–12) and great grey owls (Sn 1–4) submitted, grouped by detected infectious agent(s), the Netherlands, 1 August–13 September

	Blackbird			Owl	
	Tm 1–3,5	Tm 4,7,8,11,12	Tm 6,9,10	Sn 1–3	Sn 4
Infectious agent(s) detected^a^	Only Plasmodium	Plasmodium and USUV	Only USUV	Plasmodium and USUV	Only USUV
**Gross lesions** ^b,c^					
Hepatomegaly	4/4	3/5	1/3	2/3	1/1
Splenomegaly	4/4	4/5	3/3	3/3	1/1
Lung hyperaemia, oedema	2/4	3/5	1/3	2/3	1/1
Heart abnormalities	2/4 (1 haemopericardium, 1 pale)	1/5 (1 pale)	0/3	1/3 (1 hydropericardium)	0/1
Skin by cloaca firm, crusty	2/4	5/5	1/3	0/3	0/1
Feather abnormalities	0/4	2/5 (1 rfsh, 1 blood pens)	2/3 (2 featherless heads)	0/3	0/1
**Histological lesions** ^b,c^					
Encephalitis	0/3	2/4 (1 pvc, 1 gli/deg/pvc/swe)	1/3 (1 pvc)	0/3	1/1 (1 mix/gli)
Myocardial degeneration	0/4	1/5	1/3	0/3	1/1
Myocarditis	3/4 (1 het, 2 lym, 1 pvc)	3/5 (1 pvc/swe, 2 lym/int/ ± pvc)	2/3 (1 lym, 1 nec)	0/3	0/1
Pneumonia	3/4 (3 mix)	3/5 (2 lym/int, 1 mix)	2/3 (2 lym/int)	3/3 (2 het, 1 mix)	1/1 (1 lym/int/nec)
Kidney epithelial necrosis	1/3	4/5	1/3	0/3	0/1
Hepatitis	4/4 (4 mix)	4/5 (4 mix ± nec)	3/3 (1 lym/nec, 2 mix)	2/2 (1 mix/nec, 1 het)	1/1 (1 nec)
Splenitis	2/3 (2 mix)	4/5 (3 mix, 1 nec)	1/2 (1 lym/nec)	3/3 (1 mix/nec, 2 nec)	1/1 (1 nec)
Haemosiderosis	2/4	4/5	1/3	2/3	0/1
Skin cloaca dermatitis	2/4 (2 mix)	4/4 (2 mix, 2 lym)	1/2 (1 lym)	0/3	0/1

Deg: degeneration of white matter; gli: satellitosis, gliosis; het: heterophilic infiltrates; int: interstitial infection; lym: lymphoplasmacytic infiltrates (lymphocytes, plasma cells, histiocytes); mix: mixed infiltrates; nec: necrosis; pvc: perivascular cuffing; rfsh: retained feather shafts; Sn: *Strix nebulosa*; swe: endothelial cell swelling; Tm: *Turdus merula*; USUV: Usutu virus.

^a^ *Plasmodium* infection was determined by cytology and histology, USUV infection by RT-PCR test on brain, spleen, heart and/or liver.

^b^ Number of cases positive/total number of cases examined.

^c^ Incidental findings included gastrointestinal worms in eight of 12 blackbirds and a mycotic infection in the glandular stomach of one owl.

**Figure 1:**
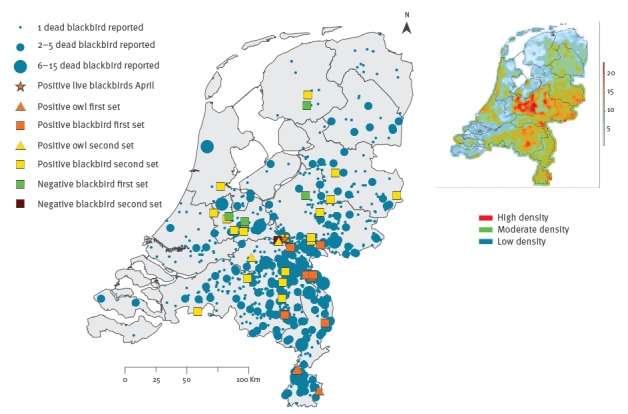
Spatial distribution of the common blackbird and great grey owl specimens tested for Usutu virus infection and common blackbird mortality as reported by the public, the Netherlands, 1 August–23 September 2016 (inset: common blackbird density 2013–15)

Initially, based on the presence of *Plasmodium* spp. schizonts and mixed inflammatory infiltrates in mainly liver and spleen, avian malaria was diagnosed (Tm 1–3, 5) [3,4]. However, when birds had myocardial degeneration (Tm 4) or encephalitis (Tm 7–8, Sn 4), tissues were submitted for USUV RT-PCR. USUV was detected in eight of 12 blackbirds (Tm 4, 6–12) and all four great grey owls (Table 2). USUV-positive cases came from sites located in the south-east of the Netherlands (Figure 1, first set). Public health authorities were informed of the outbreak, followed by a press release to inform the public on 15 September 2016.

First set: the birds examined post mortem from 1 August to 13 September 2016; second set: those obtained from 14 to 23 September 2016.

### Scale of the outbreak (second set of birds)

To gain insight in the spatial distribution of the USUV outbreak, more information was collected on deaths among blackbirds and great grey owls outside the initially identified area of USUV activity (south-east of the Netherlands). The number of reported dead blackbirds per location was extracted from reports by the public to Sovon or the Dutch Wildlife Health Centre from 1 August to 23 September 2016 and mapped using ArcGIS software by Esri (Figure 1). To visually compare this with the blackbird population density, a species distribution model was made based on more than 10,000 standardised five-minute bird counts performed during the breeding seasons from 2013 to 2015, according to a fixed grid and a large set of explanatory variables [5] (Figure 1 inset). A selection of dead blackbirds and great grey owls notified for submission by the public or owl owners between 14 to 23 September were collected for USUV testing. The selection was based on how fresh the carcass was and whether it was found at a location where USUV activity had not been identified before.

There were 924 citizen reports of which 226 mentioned that multiple sick or dead blackbirds had been observed. Most reports were from September (885/924, 96%) and from the provinces Noord Brabant (293/924, 32%), Gelderland (261/924, 28%) and Limburg (148/924, 16%). Between 14 and 23 September, 20 dead blackbirds and two great grey owls were collected for USUV testing. Nineteen of the blackbirds and two of the great grey owls tested positive for USUV (Figure 1, second set). These data support widespread occurrence of USUV infection in birds in the Netherlands in September 2016.

## Vector abundance

**Figure 2:**
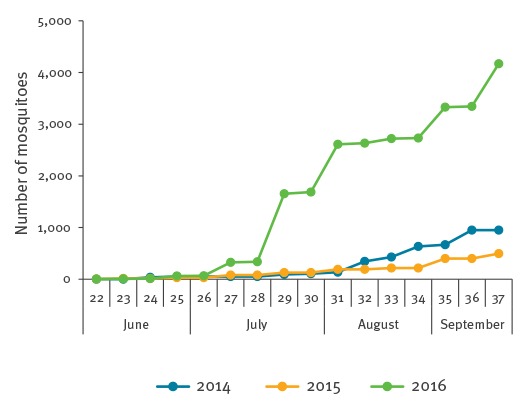
Cumulative number of mosquitoes found per year, at the sites of four used tyre companies, the Netherlands, week 22 to week 37 (end of May to mid-September)

Long-term standardised datasets on mosquito abundance are not available in the Netherlands, and arbovirus surveillance in mosquitoes is not performed. An indication of mosquito abundance in 2016 relative to previous years was obtained from data on mosquitoes found at four locations, with bi-weekly collection of mosquitoes carried out during the summer period in the years 2014 to 2016 using one trap design (BG-sentinel trap, Biogents, Germany) at sites where no insecticide treatment was applied. The total number trapped across sites in 2016 (n = 25,693) was approximately six times greater than in 2014 (n = 4,558) and approximately 10 times greater than in 2015 (n = 2,615) (Figure 2). None of the mosquito samples were tested for USUV.

The tyre companies were located throughout the country: Leeuwarden (north), Amsterdam (west), Tilburg (south) and Nederweert (east).

## Discussion

There is a widespread USUV outbreak in wild blackbirds and captive great grey owls in the Netherlands. Although USUV circulated in neighbouring countries, it had not been detected in the Netherlands before 2016, despite scanning surveillance for bird mortality since 2008 and a targeted study in dead blackbirds based on convenience sampling in 2012 [1]. USUV emerged in Europe in Italy 20 years ago [6]; however, introductions from Africa probably started several decades earlier and continue to occur [7]. The virus has been detected in mosquitoes, birds and bats in eight European countries (Austria, Belgium, Czech Republic, Germany, Hungary, Italy, Spain, Switzerland) [7,8] and is presumably maintained in enzootic mosquito–bird transmission cycles. Birds of 14 orders can be infected [8]. In the current outbreak in the Netherlands, live bird monitoring showed the presence of the virus in wild birds already months before the detection of unusual death rates among blackbirds and great grey owls. USUV outbreaks also occurred in birds in neighbouring countries, Belgium and Germany, in 2016 (personal communication: M. Garigliany and J. Schmidt-Chanasit, August 2016). A comprehensive genetic study including strain data from affected neighbouring countries is underway to elucidate the origin of events and patterns of spread.

High mosquito abundance may have been one of the factors contributing to the occurrence and scale of the outbreak in the Netherlands. In Europe, the *Culex pipiens* mosquito is considered an important vector for USUV [9,10]. The *Culex pipiens/torrentium* complex is found throughout the Netherlands between April and October [11]. June 2016 was extremely wet and, together with unusually high temperatures in September, may have furthered and prolonged mosquito activity [12,13]. The event demonstrates the need for long-term standardised datasets on mosquito abundance in the Netherlands and their analysis in relation to climate. The samples of captured mosquitoes could be one pillar in a molecular surveillance programme for USUV and other mosquito-borne zoonotic viruses.

In birds, fatal infections occur mostly in *Passeriformes* and *Strigiformes* [9,14-17]. Hepatosplenomegaly is a common finding. Histological lesions include encephalitis and necrosis in heart, liver, spleen and kidney, with lymphoplasmacytic inflammation [9,14-17]. In this outbreak, the pathological findings raised two questions. Firstly, many of the birds were co-infected with *Plasmodium* spp. Mosquitoes are the vectors of both USUV and *Plasmodium* spp., which may explain the high number of dual infections. Alternatively, a fatal outcome of USUV infection may be more probable in co-infection. Secondly, while skin lesions during USUV outbreaks have been reported earlier [9,18], causal association is unknown and needs to be studied.

We used citizen science data to identify the area where the virus probably circulated most intensively up to 23 September 2016. Infected blackbirds maintain virus circulation [15], and the observed pattern will partly reflect the density of resident blackbird populations. Ongoing wild bird counts will provide insight into the impact of USUV on resident bird populations.

The emergence of USUV in the Netherlands illustrates the continuous geographical expansion of zoonotic arboviruses in Europe, documented elsewhere [8]. It serves as another warning of the expanding geographical range of regions suitable for sustained arbovirus circulation. In areas with endemic circulation, human infections seem to occur very rarely with only 13 human cases described in literature until now [19]. Human clinical cases present with neurological signs, fever, rash, jaundice or combinations thereof. Subclinical human USUV infections are a concern in blood transfusions or organ transplants [20], and recent data from Italy suggest that subclinical cases in regions with sustained USUV circulation may be more common than previously thought [19]. The same study showed that USUV was the cause of previously unexplained encephalitis cases [19], indicating that USUV should be included in the differential diagnosis of such cases in endemic areas. These recent public health findings suggest that USUV diagnostic capability and adequate USUV surveillance with molecular typing are warranted in regions shown to be suitable for USUV circulation. Although the 2016 mosquito season is coming to an end, physicians should be aware of putative USUV infection in cases of viral encephalitis of unknown aetiology, and vigilance should be maintained in the coming mosquito season in 2017.
